# Oiled Paper, as an Economical Substitute for Oiled Silk in Surgical Dressings

**Published:** 1862-07

**Authors:** 


### OILED PAPER, AS AN ECONOMICAL SUBSTITUTE FOR OILED SILK IN SURGICAL DRESSINGS.

During a visit to England and Scotland, in the summer of 1860, I noticed in the Glasgow Royal Infirmary that they used an *oiled paper* as a substitute for oiled silk, in surgical dressings. The article was invented by Dr. McGhie, the Superintendent of the Infirmary, and possessed many advantages besides that of being economical.

The following is the mode of preparation:—Take good "tissue" paper, free from holes, as many sheets as may be required; boiled linseed oil, say one quart; to which add one ounce of sulphate of zinc, and re-boil for an hour or longer. A little beeswax and turpentine may be added, while the oil is hot. Use a square board, larger than the sheet of paper. Coat the first sheet on *both* sides with a paint or paste-brush; the rest of the sheets only require to be coated on one side, as the oil strikes through. Place the second sheet on the top of the first, slightly projecting at one end, for convenience of lifting, and so on, *seriatim*. When all the sheets are coated, hang them up to dry in a moderately warm place, for twenty-four hours. When taken down, each sheet may be dusted over with French chalk, which will prevent them from adhering. If sufficient wax and turpentine have been used in the mixture, the chalk dusting will not be needed.

Dr. McGhie, in his pamphlet, claims the following advantages for oiled paper as compared with silk:—

1. *Economy.*—A sheet costs from one to two cents only.

2. *Transparency and lightness.*—Applied over a stump or other cut surface, when hemorrhage may be feared, the state of the part can be more readily seen. On account of its lightness, it is particularly useful in covering extensive burns.

3. *Adaptability.*—It can be nicely applied to any part, retaining the form impressed upon it. It is easily torn, while, at the same time, it can be made of any required strength by doubling or trebling it.

4. *Safety.*—The great objection to oiled silk (or even to gutta percha sheeting), is, that the expense tempts us to use it over and over again; and in this way disease is propagated. There would exist no such temptation with oiled paper, as it could only be used *once*, and all risk of contagion in this way would be avoided.—*Boston Med. & Surg. Journal, Feb.* 20, 1862.

